# New Appliances and Things Medical

**Published:** 1909-05-08

**Authors:** 


### NEW APPLIANCES AND THINGS MEDICAL.

[We shal be g'ad to receive at our Office, 28 & 29 Southampton Street, Strand, London, W.C., from the manufacturers, specimens of all new preparations and appliances.]

### REGULIN.

(Chemische Fabrik Helfenberg, A. G. London Agents: Matthael and Co., 12 and 13 Cullum Street, E.C.)

We have received a sample of this laxative medicine. Two forms of it have been submitted to us. The original preparation is in the form of gum-like scales and tablets. This substance, as is well known, consists of agar-agar or so-called Japanese isinglass, which is really the dried jelly of *gelidium corneum*, a species of seaweed, to which has been added a small quantity of some purgative extract, probably cascara sagrada. Regulin acts by virtue of the fact that when it is submitted to the moisture of the intestine it swells up into a thin jelly, and thus increases the volume and softens the consistence of the fæces. In addition to this, the purgative principles it contains slightly stimulate peristalsis. It is quite tasteless, and can be taken in doses of from one teaspoonful to two tablespoonfuls. The best method of administering it is in stewed fruit or cooked vegetables, to which it should be directly added in the above quantities. The tablets, three to six of which is a dose, should be eaten directly after a meal or swallowed or partly masticated and washed down with water. They are flavoured with sugar and chocolate, and the taste of them is quite good. Regulin can be taken quite regularly without any diminution in its activity. There have been many imitations of it put on the market, but it is only fair to say that the substance made by the above firm is the original one.

---

We regret to announce the recent death of Dr. Gerald Francis Yeo, M.D., F.R.S., F.R.C.S., Emeritus Professor of Physiology at King's College, London. Professor Yeo was born in 1845, and was educated at Trinity College, Dublin, where he obtained the M.B. degree in 1867. He subsequently studied physiology in Paris, Berlin, and Vienna, and in 1874 was appointed Professor of Physiology at King's College. He occupied this important Chair for sixteen years with much distinction, and the high esteem in which he was held by contemporary physiologists is further shown by the fact that he was for fifteen years hon. secretary to the Physiological Society. For a short time in the earlier part of his career he had practised as a surgeon in Dublin, and from 1871 to 1874 he taught anatomy in the University of that city, but his tastes and abilities soon led him towards the exclusive study of the science with which his name is associated. His principal published work was a "Handbook of Physiology." Professor Yeo retired from active work some years ago, and spent his later days in South Devon. He was an enthusiastic yachtsman.

